# Pediatric esophagogastroduodenoscopy in china: indications, diagnostic yield, and factors associated with findings

**DOI:** 10.1186/s12887-022-03558-x

**Published:** 2022-09-02

**Authors:** Shengnan Wang, Xiaoxia Qiu, Jingfang Chen, Hong Mei, Haiyan Yan, Jieyu You, Ying Huang

**Affiliations:** 1 Department of Gastroenterology, Children’s Hospital of Fudan University, Shanghai, China; 2 Department of Gastroenterology, Children’s Hospital of Fudan University Xiamen Branch, Xiamen, China; 3 Department of Gastroenterology, Wuhan Children’s Hospital, Wuhan, China; 4 Department of Gastroenterology, Henan Children’s Hospital (Zhengzhou Children’s Hospital), Zhengzhou, China; 5 Department of Gastroenterology, Hunan Children’s Hospital, Changsha, China

**Keywords:** Endoscopic yield, Histologic yield, Abdominal pain, Persistent vomiting, Regression analyses

## Abstract

**Background:**

Large-scale data on esophagogastroduodenoscopy (EGD) in China are scarce. This study aimed to assess the indications and diagnostic yield of EGD in children and the relationship between factors (such as age, sex, and indications) and diagnostic yield.

**Methods:**

We performed a prospective cross-sectional observational study involving patients aged < 18 years who underwent diagnostic EGD. The study was conducted in five children’s hospitals, each in a different city. Demographic features, indications for endoscopy, and endoscopic and histopathological findings were collected. Univariable and multivariable ordinal logistic regression analyses of the relationship between the factors and diagnostic yield were performed.

**Results:**

The study included 2268 patients (male/female ratio, 1.3:1) with a median age of 8.68 years. Among the 2268 children, the most frequent indications were abdominal pain in 1954 (86.2%), recurrent vomiting in 706 (31.1%), weight loss in 343 (15.1%), and others. The endoscopic yield was 62.5% and was the highest in patients with dysphagia (90.9%). The histologic yield was 30.4% and was the highest in patients with unexplained anemia (45.5%). On multivariable regression analysis, the endoscopic yield was associated with dysphagia, gastrointestinal (GI) bleeding, and recurrent vomiting, and the histologic yield was associated with age. Different groups of patients with abdominal pain had variable probabilities of abnormal endoscopic findings.

**Conclusions:**

The most frequent indication of pediatric EGD is abdominal pain, with variable probabilities of abnormal endoscopic findings in different groups. Endoscopic yield and histologic yield are associated with certain alarming features.

**Trial registration:**

The trial registration number (ClinicalTrials. gov): NCT03603093 (The study was registered on 27/07/2018).

## Introduction

Pediatric esophagogastroduodenoscopy (EGD) began in the 1970s. Parallel to the growth of pediatric gastroenterology, an increase in the use of EGD has been observed [1]. Currently, EGD is a sensitive diagnostic tool with rare complications, which can be performed at any age [2].

EGD in children can either be diagnostic or therapeutic. In 1996, the North American Society of Pediatric Gastroenterology and Nutrition provided general indications for diagnostic upper gastrointestinal (GI) endoscopy in infants, children, and adolescents, which included the presence of symptoms indicative of an underlying organic pathology of the gastrointestinal tract [3]. American Society for Gastrointestinal Endoscopy (ASGE) provided modified guidelines for pediatric EGD in 2000, 2008, and 2014 [4–6]. In 2015, the European Society for Pediatric Gastroenterology Hepatology and Nutrition (ESPGHAN) and the European Society of Gastrointestinal Endoscopy (ESGE) developed the latest guidelines for pediatric gastrointestinal endoscopy, where the indications for diagnostic EGD were clearly stated [7].

Multiple studies have discussed the indications and diagnostic yield of EGD. Miele et al. found that nearly one-fourth of endoscopic procedures performed were inappropriate [8]. Compliance with published guidelines is associated with improved diagnostic efficiency [9]. A systematic review found that the diagnostic yield of appropriate endoscopies was higher than that of inappropriate ones (43.3% vs. 35.1%) [10]. A recent retrospective study in children found that 47.2% of upper GI endoscopies revealed abnormal findings and that age < 60 months, abdominal pain, dysphagia/odynophagia, and heartburn were predictive of abnormal endoscopy findings [11]. Another study showed that EGD was both macroscopically and histologically normal in 80.6% of cases and that unless there are alarming symptoms, younger children do not need EGD. However, studies on pediatric EGD are limited and are mostly retrospective. Based on our prior work in one center, we conducted this multicenter cross-sectional observational study to delineate the indications and diagnostic yield of EGD and depict the relationship between factors and endoscopic and histological diagnostic yields in pediatric patients who underwent their first diagnostic endoscopy.

## Methods

### Study design

This was a prospective, cross-sectional observational study (ClinicalTrials. gov ID: NCT03603093) in five hospitals, each in a different city in China. These centers were the Children’s Hospital of Fudan University in Shanghai, Children’s Hospital of Fudan University Xiamen Branch in Xiamen, Henan Children’s Hospital (Zhengzhou Children’s Hospital) in Zhengzhou, Wuhan Children’s Hospital in Wuhan, and Hunan Children’s Hospital in Changsha. Ethics approval was obtained from all five hospitals.

At the onset of this study, a steering committee was formed, comprising five directors of the gastroenterology department and 1–2 doctors in charge of data collection from each study center. A case report form (CRF) with inclusion and exclusion criteria, questionnaire, study definitions, and guidelines for data collection were provided to the centers. The questionnaire included the basic characteristics of the patients and indications for EGD. Professor Ying Huang and attendings in the Department of Gastroenterology in the Children’s Hospital of Fudan University designed the CRF and questionnaire based on their previous work.

### Patient selection and data collection

Patients aged 0–18 years who underwent diagnostic EGD in any of the five children’s hospitals were included in our study. The indications for EGD were based on the latest guidelines by ESPGHAN and ESGE. Patients with insufficient clinical data for the study, those who could not complete the EGD examinations, and those who did not conform to the defined diagnostic indications, such as removal of foreign body and endoscopic surgery were excluded from the study.

Recruitment commenced in the five selected hospitals from December 2018 to September 2019. Of the patients who underwent EGD during this period, 2268 fulfilled the inclusion criteria and gave consent to provide data on variables in the questionnaire.

Doctors responsible for data collection administered the questionnaire either to the parents of the patients or the patients themselves. Questions were about the patient’s basic characteristics, indications for EGD, and date of EGD. In addition, endoscopic and pathologic reports were combined with the questionnaires. Biopsy was performed routinely, except in those who declined biopsy.

### Indication

Indications were classified as abdominal pain, recurrent vomiting, weight loss or failure to thrive, GI bleeding, unexplained anemia, symptoms of gastroesophageal reflux (GER), diarrhea, caustic ingestion, dysphagia, etc., according to the latest guidelines by ESPGHAN and ESGE. Some patients had more than one indication.

### Endoscopic and histologic findings

A positive endoscopic yield was defined as the presence of relevant findings on endoscopy, grouped into categories such as esophagitis, gastritis, duodenitis, peptic ulcer, *Helicobacter pylori* infection diagnosed through rapid urease test, and others. The histologic findings mainly included moderate or severe inflammation or *Helicobacter pylori* infection confirmed by immunohistochemical staining.

### Data management

The questionnaires and endoscopic and pathologic reports from the other four centers were sent back to the center in Shanghai. Data entry was performed by two people using the EpiData software. After double entry, the data were compared; non-conforming data were confirmed by referring to the original questionnaire.

### Statistical analysis

All analyses were performed using Stata 12.0 statistical software (StataCorp LP, College Station, TX, USA). Descriptive statistics were calculated for demographic features (sex and age). After generating descriptive counts and proportions for symptom variables, Chi-square tests were used to compare patients with different demographic features or symptom variables for diagnostic yield. Then, univariable and multivariable logistic analyses were performed to assess the relationship between demographic features or symptom variables and the presence of a positive endoscopic or histologic abnormality. Significance was set at *p* < 0.05.

## Results

### Study center characteristics

During the study period, 2287 questionnaires were administered. Those that did not conform to the indications and those with missing data were discarded. Eventually, 2268 questionnaires were included in the analyses, comprising 914 questionnaires from Shanghai (population: 24.28 million), 500 from Wuhan (population: 11.21 million), 382 from Zhengzhou (population: 10.35 million), 337 from Xiamen (population: 4.29 million), and 135 from Changsha (population: 8.39 million). The mean age (SD) of the participants was 8.68 years (3.25), and 57% of them were male (Table 1).

**Table 1:** Baseline patient characteristics

Characteristics	
Total	2268
Shanghai	914
Wuhan	500
Zhengzhou	382
Xiamen	337
Changsha	135
Male/female	1.3:1 (1294/974)
Age (IQR)	8.68 ± 3.25 (0.28–17.58)
0–1 y	16
1–5 y	292
5–12 y	1575
Teenagers (13–18 y)	385
Biopsies	
Yes	2235
No	34

### Indications

Overall, the common indications for endoscopy were abdominal pain (86.2%), persistent vomiting (31.1%), weight loss (15.1%), GER symptoms (8.4%), GI bleeding (4.7%), diarrhea (3.7%), unexplained anemia (0.5%), and dysphagia (0.7%). Many patients had over one recorded indication. The indications were similar in different centers, i.e., abdominal pain was the most common indication.

### Endoscopic findings and yield

Some patients had more than one endoscopic finding. The overall prevalence of abnormal endoscopic findings was 62.5%; abnormal findings were found in the esophagus (6.7%), stomach (30.2%), and duodenum (21.6%). The rapid urease test was positive in 32.8% of the 2097 patients. Table 2 shows the differences in endoscopic yield between patients of different age groups or symptom variables. Compared with male patients, female patients had a similar rate of abnormal endoscopic findings. A significant difference was found in the endoscopic yield among the four age groups; infants and teenagers aged 13–18 years were much more likely to have abnormal endoscopic findings than participants in other age groups (*p* < 0.05). The endoscopic yield was the highest in patients with dysphagia (94.1%), followed by unexplained anemia (90.9%), GI bleeding (83.0%), weight loss (68.5%), recurrent vomiting (66.9%), GER symptoms (66.5%), diarrhea (66.3%), and abdominal pain (62.5%).

**Table 2:** Patient characteristics according to endoscopic and histologic findings

Variable	Total patients *n* = 2268 (%)	With endoscopic findings *n* = (%)	P value	With histologic findings *n* = (%)	P value
Sex					
Male	1294	808 (62.4%)	0.928	368 (28.8%)	0.875
Female	974	610 (62.6%)		279 (29.1%)	
Age					
0–1 y	16	13 (81.3%)	0.001	5 (41.7%)	0.008
1–5 y	292	183 (62.7%)		64 (22.3%)	
5–12 y	1575	951 (60.4%)		449 (28.8%)	
Teenagers (13–18 y)	385	271 (70.4%)		129 (34.0%)	
Abdominal pain					
+	1954	1215 (62.5%)	0.401	536 (27.7%)	0.001
-	314	203 (64.7%)		111 (37.1%)	
Recurrent vomiting					
+	706	472 (66.9%)	0.004	185 (26.9%)	0.468
-	1562	946 (60.6%)		462 (30.0%)	
Weight loss					
+	343	235 (68.5%)	0.013	112 (32.9%)	0.078
-	1925	1183 (61.5%)		535 (28.2%)	
Gastroesophageal reflux symptoms					
+	191	127 (66.5%)	0.236	69 (36.3%)	0.019
-	2077	1291 (62.2%)		578 (28.3%)	
Gastrointestinal bleeding					
+	106	88 (83.0%)	0.000	35 (35.0%)	0.172
-	2162	1330 (61.5%)		612 (28.7%)	
Diarrhea					
+	83	55 (66.3%)	0.473	24 (28.9%)	0.995
-	2185	1363 (62.4%)		623 (29.0%)	
Unexplained anemia					
+	11	10 (90.9%)	0.051	5 (45.5%)	0.226
-	2257	1408 (62.4%)		642 (28.9%)	
Dysphagia					
+	17	16 (94.1%)	0.007	4 (25.0%)	0.727
-	2251	1402 (62.3%)		643 (29.0%)	

The results of the logistic regression analyses of the symptom variables associated with endoscopic yield are shown in Table 3. On univariate analysis, the endoscopic yield was associated with dysphagia, GI bleeding, weight loss, and recurrent vomiting. On multivariable analysis, dysphagia (*p* < 0.05), GI bleeding (*p* < 0.01), and recurrent vomiting (*p* < 0.05) were independently associated with endoscopic yield.

**Table 3:** Univariate and multivariate regression analyses of endoscopic findings

Variable		Univariate analysis				Multivariate analysis		
	OR		95% CI	P value	OR		95% CI	*P* value
Age	1.02		0.99–1.05	0.125				
Sex	1.01		0.85–1.20	0.928				
Abdominal pain	0.9		0.70–1.15	0.402				
Recurrent vomiting	1.31		1.09–1.58	0.004	1.21		1.00–1.47	0.046
Weight loss	1.36		1.07–1.74	0.013	1.27		0.99–1.64	0.057
Gastroesophageal reflux symptoms	1.21		0.88–1.65	0.237				
Gastrointestinal bleeding	3.06		1.83–5.12	0.000	2.85		1.70–4.79	0.000
Diarrhea	1.18		0.75–1.88	0.473				
Unexplained anemia	6.03		0.77–47.19	0.087				
Dysphagia	9.69		1.28–73.19	0.028	9.61		1.27–72.7	0.028

### Pathologic findings and yield

Biopsy samples were obtained from 2235 patients and pathologically examined. The overall prevalence of histological abnormalities was 30.4%. Table 2 shows the differences in histologic yield between patients of different age groups or symptom variables. Compared with male patients, female patients had a similar rate of occurrence of histologic abnormalities. A significant difference in histologic yield was found among the four age groups, with infants most likely to have histological abnormalities (*p* < 0.05). The histologic yield was the highest in patients with unexplained anemia (45.5%), followed by GER symptoms (36.3%), GI bleeding (35.0%), weight loss (32.9%), diarrhea (28.9%), abdominal pain (27.7%), recurrent vomiting (26.9%), and dysphagia (25%).

The results of the logistic regression analyses of the symptom variables associated with histologic yield are shown in Table 4. On univariate analysis, the histologic yield was positively associated with factors such as age and gastroesophageal reflux symptoms. Patients who had the indication of abdominal pain appeared to have less likelihood of histologic abnormality than those without. On multivariable analysis, age (*p* < 0.05) remained positively correlated with histologic yield.

**Table 4:** Univariate and multivariate regression analyses of histologic findings

Variable	Univariate analysis			Multivariate analysis		
	OR	95% CI	P value	OR	95% CI	P value
Age	1.27	1.08–1.50	0.004	1.30	1.10–1.53	0.002
Sex	1.01	0.84–1.22	0.875			
Abdominal pain	0.65	0.50–0.84	0.001	0.62	0.48–0.81	0.000
Recurrent vomiting	0.86	0.70–1.05	0.144			
Weight loss	1.25	0.98–1.60	0.078			
Gastroesophageal reflux symptoms	1.45	1.06–1.98	0.020	1.33	0.97–1.82	0.076
Gastrointestinal bleeding	1.34	0.88–2.04	0.174			
Diarrhea	1.00	0.62–1.62	0.995			
Unexplained anemia	2.05	0.62–6.75	0.236			
Dysphagia	0.82	0.26–2.54	0.727			

### The indication of abdominal pain

A significant difference was found in the endoscopic yield among the patients with abdominal pain (Table 5). In these patients, those with any other additional symptoms such as vomiting, weight loss, GER symptoms, gastrointestinal bleeding, diarrhea, unexplained anemia, and dysphagia were much more likely to have abnormal endoscopic findings than those without (*p* < 0.05). Additionally, patients with abdominal pain and greater than three other symptoms had the highest rate of abnormal endoscopic findings than other groups (*p* < 0.05). Patients with abdominal pain and evidence of *Helicobacter pylori* infection or with family members having *Helicobacter pylori* infection were much more likely to have abnormal endoscopic findings than those without (*p* < 0.05).

**Table 5:** Endoscopic findings in different groups of patients with abdominal pain

Groups	With endoscopic findings, *n* = (%)	*P* value
Without any other symptoms	647 (58.8%)	
With any other symptoms	568 (66.6%)	0.000
1^*^	380 (64.0%)	
2^*^	138 (68.0%)	
3^*^	50(89.3%)	0.001
With Helicobacter pylori infection evidence		
+	209 (87.8%)	
-	1006 (58.6%)	0.000
With family members with Helicobacter pylori infection		
+	225 (68.6%)	
-	990 (60.9%)	0.009

^*^The number represents the number of symptoms that have occurred

## Discussion 

Pediatric EGD aids the understanding of the pathophysiology of common GI disorders in children and plays an important role in the management of some disorders. Parallel to the utilization of endoscopy in pediatric patients, the volume of EGD being performed has increased. When using this tool, we need to review its use to maximize its efficacy.

In this multicenter study, we found that abdominal pain was the most common indication, followed by recurrent vomiting, weight loss, GER symptoms, GI bleeding, diarrhea, and others. According to studies performed in large children’s hospitals or pediatric clinics, abdominal pain is also the most common indication for upper GI endoscopy in UK and US cohorts [12–14]. A retrospective analysis (carried out over 20 years from 1985 to 2005) of children and adolescents who underwent EGD at a single center revealed that the proportion of patients with abdominal pain increased from 23 to 43%, while that of patients with GI bleeding declined from 34 to 5% over the 20-year interval [1]. Studies in some small countries have found the most frequent indication in their centers was surveillance for esophageal varices and suspected celiac disease [15–17].

Currently, guidelines provide the indications for EGD; the guidelines developed and newly modified in 2014 by ASGE provide indications for EGD [6]. The guidelines developed in 2015 by ESPGHAN and ESGE also elaborate indications for EGD [7]. These two guidelines have the following in common: abdominal pain, weight loss, failure to thrive, unexplained anemia, dysphagia or odynophagia, caustic ingestion, recurrent vomiting with unknown cause, GI bleeding, diarrhea/malabsorption (chronic), and intractable or chronic symptoms of GERD. However, unexplained irritability, anorexia, and suspicion of graft versus host disease differentiate them.

In previous retrospective studies, the diagnostic yield of EGD was significantly different, varying from 18.9 to 79% [13–18]. Several more recent studies have identified certain basic patient characteristics including age that affect diagnostic yield. The diagnostic yield was found to be higher in teenagers [11, 13, 14, 19] and lower in those aged < 7 years, especially in those without alarming symptoms [20]. In this study, we found that the infant group and the teenager group appeared to have a higher yield of abnormal endoscopic and histological findings, similar to the results of previous studies [11, 13, 14, 19].

Except for the basic characteristics, the composition of the patients in the study also affected the overall diagnostic yield, because diagnostic yield varies in patients with different indications for EGD. In our multivariate regression analyses, we observed that abdominal pain was the most common indication; however, patients with abdominal pain had a lower rate of abnormal endoscopic or histological findings than those without. This finding is similar to that of a large retrospective study of 1,000 children in 2013, in which the most common indication (abdominal pain) had lower rates of abnormal endoscopic findings (28.9%) than other indications including stricture which was confirmed on upper GI series (100%), foreign body (88%), GI bleeding (57%), dysphagia (56%), and positive celiac screening (52%) [14]. Other studies have also shown that patients with generalized abdominal pain had a lower rate of abnormal endoscopic findings (36%) than those with UGI bleeding (71.3%), variceal surveillance (54.8%), recurrent persistent vomiting (38%), and dyspepsia (37.8%) [17].

Our study findings can be a further supplement to the indication of abdominal pain. Our study showed that patients with indications of abdominal pain and any other symptoms (such as recurrent vomiting, weight loss or failure to thrive, GI bleeding, unexplained anemia, GER symptoms, diarrhea, caustic ingestion, and dysphagia), those with evidence of *Helicobacter pylori* infection before EGD, or those whose family members had *Helicobacter pylori* infection had higher rates of abnormal endoscopic findings. Therefore, these patients seemed to have more necessity to receive EGD.

Biopsy is often performed during diagnostic endoscopy to determine the pathology of focal lesions or identify the presence of *Helicobacter pylori*. The guidelines developed by ESPGHAN and ESGE in 2015 recommend routine tissue sampling even in the absence of visible endoscopic abnormalities in all children undergoing EGD. The low rate of abnormal endoscopic findings in our study may be due to the presence of mild chronic inflammation of the mucosa in most patients.

Our study has few limitations, which could be attributed to the multicenter nature of the study. Although prior training of the endoscopists and investigators were conducted regarding the methodology to ensure uniformity, heterogeneity could not be completely eliminated. Moreover, heterogeneity might also exist due to the difference in pathological diagnosis by pathologists at different centers. Hence, the indications and diagnostic yields of gastroscopy should be validated in future evidence-based studies for better patient management.

In conclusion, EGD is valuable for diagnosis in children with digestive symptoms, especially those with alarming features such as dysphagia, GI bleeding, and recurrent vomiting. Biopsy and histological examinations should be performed more aggressively in infants, those with weight loss or without abdominal pain.

## Data Availability

The datasets used and analyzed during the current study are not publicly available due not all of the researchers wish to share the data with public at present, but available from the corresponding author on reasonable request.

## Abbreviations

EGD: Esophagogastroduodenoscopy
GI: Gastrointestinal
ASGE: American Society for Gastrointestinal Endoscopy
ESGE: European Society of Gastrointestinal Endoscopy
ESPGHAN: European Society for Pediatric Gastroenterology Hepatology and Nutrition
CRF: Case report form
GER: Gastroesophageal reflux
CAP: Chronic abdominal pain

## References

[1] Franciosi JP, Fiorino K, Ruchelli E, Shults J, Spergel J, Liacouras CA (2010). Changing indications for upper endoscopy in children during a 20-year period. J Pediatr Gastroenterol Nutr.

[2] Ament ME, Berquist WE, Vargas J, Perisic V (1988). Fiberoptic upper intestinal endoscopy in infants and children. Pediatr Clin North Am.

[3] Squires RJ, Colletti RB (1996). Indications for pediatric gastrointestinal endoscopy: a medical position statement of the North American Society for Pediatric Gastroenterology and Nutrition. J Pediatr Gastroenterol Nutr.

[4] Eisen GM, Chutkan R, Goldstein JL, Petersen BT, Ryan ME, Sherman S (2000). Modifications in endoscopic practice for pediatric patients. Gastrointest
Endosc.

[5] Lee KK, Anderson MA, Baron TH, Banerjee S, Cash BD, Dominitz JA (2008). Modifications in endoscopic practice for pediatric patients. Gastrointest
Endosc.

[6] Lightdale JR, Acosta R, Shergill AK, Chandrasekhara V, Chathadi K, Early D (2014). Modifications in endoscopic practice for pediatric patients. Gastrointest
Endosc.

[7] Tringali A, Thomson M, Dumonceau JM, Tavares M, Tabbers MM, Furlano R (2017). Pediatric gastrointestinal endoscopy: European Society of Gastrointestinal Endoscopy (ESGE) and European Society for Paediatric Gastroenterology Hepatology and Nutrition (ESPGHAN) Guideline Executive summary. Endoscopy.

[8] Miele E, Giannetti E, Martinelli M, Tramontano A, Greco L, Staiano A (2010). Impact of the Rome II paediatric criteria on the appropriateness of the upper and lower gastrointestinal endoscopy in children. Aliment Pharmacol Ther.

[9] Jantchou P, Schirrer J, Bocquet A (2007). Appropriateness of upper gastrointestinal endoscopy in children: a retrospective study. J Pediatr Gastroenterol Nutr.

[10] Zullo A, Manta R, De Francesco V, Fiorini G, Hassan C, Vaira D (2019). Diagnostic yield of upper endoscopy according to appropriateness: a systematic review. Dig Liver Dis.

[11] Altamimi E, Odeh Y, Al-Quraan T, Mohamed E, Rawabdeh N (2021). Diagnostic yield and appropriate indication of upper endoscopy in Jordanian children. BMC Pediatr.

[12] Wang S, Younus O, Rawat D, Naik S, Giles E, Meadows N (2018). Clinical presentation and outcomes of diagnostic endoscopy in newly presenting children with gastrointestinal symptoms. J Pediatr Gastroenterol Nutr.

[13] Lyons H, Zhang Y, Szpunar S, Dharmaraj R (2017). Predictors of positive esophagogastroduodenoscopy outcomes in children and adolescents: a single center experience. BMC Res Notes.

[14] Sheiko MA, Feinstein JA, Capocelli KE, Kramer RE (2013). Diagnostic yield of EGD in children: a retrospective single-center study of 1000 cases. Gastrointest
Endosc.

[15] Lee WS, Zainuddin H, Boey CC, Chai PF (2013). Appropriateness, endoscopic findings and contributive yield of pediatric gastrointestinal endoscopy. World J Gastroenterol.

[16] Berger TD, Soffer S, Vurzel-Harel T, Silbermintz A, Fleishaker H, Shamir R (2020). The yield of upper gastrointestinal endoscopy at a Pediatric Tertiary Care Center. Isr Med Assoc J.

[17] Wani MA, Zargar SA, Yatoo GN, Haq I, Shah A, Sodhi JS (2020). Endoscopic yield, appropriateness, and complications of pediatric upper gastrointestinal endoscopy in an adult suite: a retrospective study of 822 children. Clin Endosc.

[18] Thomson M, Sharma S (2017). Diagnostic yield of upper and lower gastrointestinal endoscopies in children in a Tertiary Centre. J Pediatr Gastroenterol Nutr.

[19] Noble AJ, Drouin E, Tamblyn R (2008). Design of predictive models for positive outcomes of upper and lower gastrointestinal endoscopies in children and adolescents. J Pediatr Gastroenterol Nutr.

[20] Helin N, Kolho KL, Rintala R, Merras-Salmio L (2020). Upper endoscopy for non-acute non-specific symptoms is seldom beneficial for children under the age of seven. Acta Paediatr.

